# Chinese SLE Treatment and Research Group Registry: III. Association of Autoantibodies with Clinical Manifestations in Chinese Patients with Systemic Lupus Erythematosus

**DOI:** 10.1155/2014/809389

**Published:** 2014-04-23

**Authors:** Jing Li, Xiaomei Leng, Zhijun Li, Zhizhong Ye, Caifeng Li, Xiaofeng Li, Ping Zhu, Zhengang Wang, Yi Zheng, Xiangpei Li, Miaojia Zhang, Xin-Ping Tian, Mengtao Li, Jiuliang Zhao, Feng-Chun Zhang, Yan Zhao, Xiaofeng Zeng

**Affiliations:** ^1^Key Laboratory of Rheumatology and Clinical Immunology, Department of Rheumatology, Peking Union Medical College Hospital, Ministry of Education, No. 1 Shuaifuyuan, Beijing 100730, China; ^2^Department of Rheumatology, The Affiliated Hospital of Bengbu Medical College, No. 2600 Do Hai Da Dao, Anhui 233030, China; ^3^Department of Rheumatology, The Fourth People's Hospital of Shenzhen Affiliated to Guangdong Medical College, No. 22 Nong Lin Lu, Guangdong 518034, China; ^4^Department of Rheumatology, Beijing Children's Hospital Affiliated to Capital Medical University, No. 56 Nan Li Shi Lu, Beijing 100045, China; ^5^Department of Rheumatology, The Second Hospital of Shanxi Medical University, No. 382 Wu Yi Lu, Shanxi 030001, China; ^6^Department of Clinical Immunology and Rheumatology, Xijing Hospital Affiliated to the Fourth Military Medical University, No. 15 Chang Le Xi Lu, Shanxi 710032, China; ^7^Department of Rheumatology, Beijing Tongren Hospital Affiliated to Capital Medical University, No. 1 Dong Jiao Min Xiang, Beijing 100730, China; ^8^Department of Rheumatology, Beijing Chao-yang Hospital Affiliated to Capital Medical University, No. 8 Gong Ti Nan Lu, Beijing 100020, China; ^9^Department of Rheumatology, Anhui Provincial Hospital, No. 17 Lu Jiang Lu, Anhui 230001, China; ^10^Department of Rheumatology, Jiangsu Provincial People's Hospital, No. 30 Guang Zhou Lu, Jiangsu 210029, China; ^11^Department of Rheumatology, Peking Union Medical College and Chinese Academy of Medical Sciences, Peking Union Medical College Hospital, Beijing 100730, China

## Abstract

We investigated the characteristics of Chinese SLE patients by analyzing the association between specific autoantibodies and clinical manifestations of 2104 SLE patients from registry data of CSTAR cohort. Significant (*P* < 0.05) associations were found between anti-Sm antibody, anti-rRNP antibody, and malar rash; between anti-RNP antibody, anti-SSA antibody, and pulmonary arterial hypertension (PAH); between anti-SSB antibody and hematologic involvement; and between anti-dsDNA antibody and nephropathy. APL antibody was associated with hematologic involvement, interstitial lung disease, and a lower prevalence of oral ulcerations (*P* < 0.05). Associations were also found between anti-dsDNA antibody and a lower prevalence of photosensitivity, and between anti-SSA antibody and a lower prevalence of nephropathy (*P* < 0.05). Most of these findings were consistent with other studies in the literature but this study is the first report on the association between anti-SSA and a lower prevalence of nephropathy. The correlations of specific autoantibodies and clinical manifestations could provide clues for physicians to predict organ damages in SLE patients. We suggest that a thorough screening of autoantibodies should be carried out when the diagnosis of SLE is established, and repeated echocardiography annually in SLE patients with anti-RNP or anti-SSA antibody should be performed.

## 1. Introduction

Systemic lupus erythematosus (SLE) is one of the most complicated autoimmune diseases. It could involve almost all organs or systems and presents with protean clinical manifestations [1]. In general, SLE can be divided into several subgroups based on specific clinical features including age, gender, and autoantibodies pattern, and the prognosis of different subgroups varies [2]. Anti-Sm antibody is considered as the marker autoantibody for the diagnosis of SLE with reported positivity ranged from 15.4% to 21.8% [3, 4]. Anti-double stranded DNA (anti-dsDNA) antibody is another specific autoantibody for SLE and has been proven to be associated with disease activity of SLE [5]. In order to understand SLE better, the association between clinical features of SLE and other anti-extractable nuclear antigen (ENA) antibodies (e.g., anti-SSA, anti-SSB, anti-RNP, and anti-rRNP antibodies) had been investigated by many research groups [6, 7]. In our study, we analyzed the associations between clinical manifestations and autoantibody patterns in Chinese SLE patients based on the data from Chinese SLE Treatment and Research group (CSTAR) registry. CSTAR is the first online registry of Chinese SLE patients and is supported by the Chinese National Key Technology R&D Program. This registry has depicted major clinical characteristics of lupus in Chinese patients [8].

## 2. Methods and Patients

### 2.1. Patients

CSTAR launched the first registry project of Chinese SLE patients in 2009, which was approved by the Institute Review Board (IRB) of Peking Union Medical College Hospital (PUMCH). Other centers had received ethical approval by the local IRB. All investigators were trained for the diagnosis, history review, disease activity evaluation, laboratory examinations, data input, and sample collection by local or nationwide training programs. This ongoing registry had recruited 2170 Chinese SLE patients who fulfilled the SLE classification criteria revised by the American College of Rheumatology (ACR) in 1997 [9] during the period between April 2009 and February 2010. Patients were required to fulfill at least 4 of the following 11 criteria: (1) malar rash; (2) discoid rash; (3) photosensitivity; (4) oral or nasopharyngeal ulceration; (5) nonerosive arthritis involving 2 or more peripheral joints; (6) pleuritis or pericarditis; (7) nephropathy: persistent proteinuria > 0.5 grams per day or cellular casts; (8) neurologic involvement: seizures or psychosis in the absence of offending drugs or known metabolic derangements; (9) hematologic involvement: hemolytic anemia with reticulocytosis or leukopenia (<4,000/mm^3^ on ≥2 occasions) or lymphopenia (<1,500/mm^3^ on ≥2 occasions) or thrombocytopenia (<100,000/mm^3^) in the absence of offending drugs; (10) immunologic disorder: antibody to native double-stranded DNA in abnormal titer or presence of antibody to Sm nuclear antigen or positive finding of antiphospholipid antibodies; (11) positive antinuclear antibody. We confirmed positive finding of antiphospholipid antibodies by an abnormal serum level of IgG or IgM anticardiolipin antibodies, an abnormal serum level of anti-*β*2 glucoprotein I, or a positive test result for lupus anticoagulant. In this study, we analyzed baseline data of 2104 patients (including 190 male and 1914 female patients) (Table 1).

### 2.2. Methods

Each center of the CSTAR has provided uniform evaluations and recorded data following the same protocol and operational procedures. Clinical manifestations and systemic involvement of SLE patients were collected, evaluated, and the relevant data were entered into the online CSTAR registry database. Pulmonary arterial hypertension (PAH) was defined as a resting systolic PAP (PASP) ≥40 mmHg estimated by echocardiography [10] without chronic lung conditions, cardiac valvular, or cardiomyopathic complications. Interstitial lung disease (ILD) was detected by chest X-ray or computerized tomography without infective infiltrations. Patients who had ILD with known causes or PAH were excluded when the association between autoantibodies and ILD in this study was analyzed.

The autoantibodies were measured at the local labs of each center, including anti-nuclear antibody (ANA), anti-double stranded DNA (anti-dsDNA) antibody, anti-Sm antibody, anti-ribosomal RNA-protein (anti-rRNP) antibody, anti-SSA antibody, anti-SSB antibody, anti-u1 small-nuclear RNA-protein (anti-RNP) antibody, and anti-phospholipid (APL) antibody. Most centers detected ANA and anti-dsDNA antibody using immunofluorescence assay with Hep-2 cell line and anti-extractable nuclear antigen (ENA) antibody (including anti-Sm, anti-SSA, anti-SSB, anti-RNP, and anti-rRNP antibodies) was tested with immunoblotting assay. The APL antibody was tested using enzyme-linked immunosorbent assay (anticardiolipin and anti-*β*2 glucoprotein I antibody) or dilute Russell viper venom test (lupus anticoagulant) when anti-phospholipid syndrome was suspected but these tests were not mandatory.

### 2.3. Statistical Analysis

Statistical analysis was performed by using SPSS 17.0 for WINDOWS. Positivity of autoantibodies in SLE patients with different clinical manifestations was expressed as patient number with percentage (%) in brackets (Table 4). Chi-square tests were performed to detect the associations between clinical manifestations and autoantibody patterns. Since there was no expected frequency <5, Fisher's exact test was not used. Cluster analysis with Ward's method was performed to investigate the relationship between autoantibodies. All tests of significance were two-sided and a *P* value of < 0.05 was considered to be statistically significant.

## 3. Results

The autoantibody profile of this study included the presence of ANA in 2063 (98.1%), anti-dsDNA antibody in 699 (33.2%), anti-Sm antibody in 350 (16.6%), anti-RNP antibody in 189 (8.9%), anti-SSA antibody in 497 (23.6%), anti-SSB antibody in 224 (10.7%), and anti-rRNP antibody in 255 (12.7%) cases. APL antibody was tested in 937 patients with a positivity of 44.1% (414/937). 199 patients (9.5%) were demonstrated to have both anti-SSA antibody and anti-SSB antibody and 142 patients (6.7%) have anti-Sm antibody and anti-RNP antibody, simultaneously (Table 2).

Clinical manifestations found in our study included malar rash in 1009 (47.9%), discoid skin lesions in 118 (5.6%), photosensitivity in 526 (25.0%), oral ulcer in 466 (22.1%), arthritis in 1147 (54.5%), serositis in 345 (16.4%), nephropathy in 988 (47.4%), hematological involvement, including leukocytopenia, hemolytic anemia, and thrombocytopenia, in 1181 (56.1%), and neurological involvement (neuropsychological lupus) in 101 (4.8%) patients. The prevalence of ILD and PAH was 4.2% (86/2024) and 3.8% (74/1934), respectively in this registry database (Table 3).

The association analysis between clinical manifestations and autoantibodies revealed that there were associations between anti-Sm antibody (*P* < 0.001), anti-rRNP antibody (*P* < 0.05), and malar rash; between anti-dsDNA antibody and nephropathy; between anti-RNP antibody, anti-SSA antibody and pulmonary arterial hypertension (PAH); and between anti-SSB antibody and hematological involvement (*P* < 0.05). Significant associations were also found between anti-dsDNA antibody and a lower prevalence of photosensitivity and between anti-SSA antibody and a lower prevalence of nephropathy (*P* < 0.05). APL antibody was associated with hematologic involvement, interstitial lung disease (ILD), and a lower prevalence of oral ulcerations (*P* < 0.05) (Table 4).

**Figure 1:**
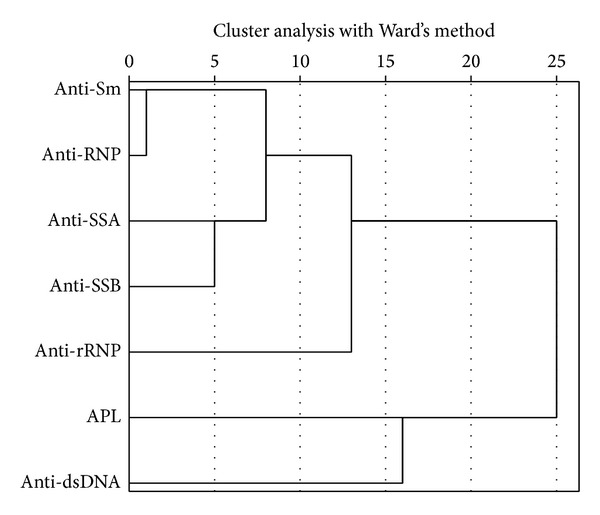
Result of cluster analysis with Ward's method in SLE patients. Five clusters of antibodies were identified. Cluster 1 consisted of antibodies to Sm and RNP and cluster 2 consisted of antibodies to SSA and SSB. Clusters 3, 4, and 5 consisted of antibodies to ribosomal P, dsDNA, and APL, respectively.

Using cluster analysis, we identified five clusters of antibodies. Cluster 1 consisted of antibodies to Sm and RNP and cluster 2 consisted of antibodies to SSA and SSB. Clusters 3, 4, and 5 consisted of antibodies to ribosomal P, dsDNA, and APL, respectively (Figure 1).

## 4. Discussion

Diffuse connective tissue diseases (CTDs) are characterized by the presentation of specific profiles of autoantibodies, and SLE is the prototype of diffuse CTDs, which could involve almost all systems [1]. The typical pathological feature of SLE is systemic vasculitis with immune complex deposition [11]. It is rational to propose that some autoantibodies may be associated with specific clinical features of SLE.

Anti-dsDNA has been proven to be a pathogenic autoantibody in SLE and has been reported to be associated with renal damage, leukopenia, anemia, and urine cellular casts in SLE patients [6, 7, 12]. Chien et al. discovered that there were associations between anti-dsDNA antibodies and multiple clinical manifestations of SLE patients, such as Raynaud's phenomenon, photosensitivity, arthritis, hypocomplementemia, thrombocytopenia, proteinuria, and serositis, but with a small number of patients (80 patients) [13]. Our study confirmed that anti-dsDNA was associated with nephropathy (Table 4) which was also reported by Lu et al. and Alba et al. [12, 14]. The association between anti-dsDNA antibody and a lower prevalence of photosensitivity observed in our study was contradictory to Smikle et al. [4]. The difference may be due to different ethnical background and sample size.

As a marker autoantibody of SLE, anti-Sm antibody was found to be associated with malar rash, discoid rash, pericarditis, and leukopenia in studies by Tang et al. and Lu et al. [7, 12] However, the pathogenic characteristics of anti-Sm antibody were controversial [6, 12, 14, 15]. Our study also revealed the association of anti-Sm antibody with malar rash, and malar rash is a characteristic skin lesion of SLE patients. But the association between anti-Sm antibody and other organ damage could not be detected in our study (Table 4).

Anti-RNP antibody was thought to be related to Raynaud's phenomenon and PAH by many physicians. The association between anti-RNP and Raynaud's phenomenon was confirmed by Hoffman et al. and Tang et al. [6, 7]. Both Raynaud's phenomenon and anti-RNP antibody are considered as risk factors for PAH associated with CTDs and represent the presence of vasculopathy [16]. Anti-RNP antibody was also found to be associated with photosensitivity [7], lymphopenia [12], and leukopenia [6]. In our study, the Raynaud's phenomenon was not included in the clinical manifestations analysis, but the association between anti-RNP antibody and PAH in patients with SLE was discovered (Table 4).

Anti-SSA and anti-SSB antibody are frequently found in SLE patients with the positivity ranged from 34% to 83% in different reports [8, 17] and higher prevalence of anti-SSA/SSB antibody could be observed in SLE patients associated with secondary Sjogren's syndrome [18]. Anti-SSA antibody was found to be associated with neonatal heart block [19], xerophthalmia/xerostomia, and photosensitivity [20]. Anti-SSB body was found to be associated with hematological disorder, proteinuria, malar rash [12], and pericarditis [6]. In this study, we found the associations between anti-SSA antibody and PAH, between anti-SSB antibody and hematological involvement, and between anti-SSA antibody and a lower prevalence of nephropathy (Table 4). This study is the first report that anti-SSA antibody might be a predictor of PAH in SLE patients according to our knowledge. Since we have known that anti-SSA antibody is one of the diagnostic criteria for Sjogren's syndrome [21] and PAH is also a rare manifestation of patients with primary Sjogren's syndrome [22], we propose that more attention should be paid to screen for PAH in SLE patients with anti-SSA antibody. But further studies are needed to clarify this association in the future. The association between anti-SSA antibody and a lower prevalence of lupus nephritis was reported by Chien and coresearchers in a small sample size study [13] and Tapanes et al. found that anti-SSA antibody may correlate with favorable prognosis of lupus nephritis [23]. But Vila et al. found the opposite results in 201 Puerto Ricans patients with SLE [24]. We confirmed the association between anti-SSA antibody and a lower prevalence of nephropathy in Chinese SLE patients and this association may suggest a protective role of anti-SSA antibody in lupus nephritis. The hematological disorder in SLE patients with positive anti-SSB antibody was primarily thrombocytopenia in our study and the association between anti-SSB antibody and thrombocytopenia was reported by Unal et al. in a case report [25]. Large sample studies are needed to clarify the real association between the hematological disorders and anti-SSB antibody.

Anti-rRNP antibody is regarded as a specific autoantibody of SLE [26] and it is thought to be associated with neuropsychological manifestations of SLE patients [27]. This relationship was not proven in our study, what may be due to the small amount of patients with neuropsychological manifestations in our registry. The association of anti-rRNP antibody and malar rash was found in our study (Table 4), which was consistent with anti-Sm antibody.

The APL antibody was tested in 937 patients when antiphospholipid syndrome was suspected in our cohort study. The association between APL antibody and hematological involvement (mainly thrombocytopenia) was deductible since phospholipid is an innate component of blood cells. McClain and coresearchers have found that APL antibody appeared prior to diagnosis of SLE and this group of autoantibodies are associated with many SLE features, including malar rash, discoid lesions, photosensitivity, renal disorder, neurological disorder, hemolytic anemia, and thrombocytopenia [28]. Saches and coresearchers have demonstrated that APL antibody is associated with spontaneous abortion, thrombocytopenia, livedo reticularis, and a positive direct Coombs' test in SLE patients [29]. The association between APL antibody and ILD found in our study may be related to the microvessel injuries resulted from microemboli or immune-complex deposition (Table 4). Kanakis et al. have reviewed the pulmonary manifestations in patients with antiphospholipid syndrome including fibrosing alveolitis [30]. We confirmed the association between antiphospholipid antibody and ILD, which is a rare feature of SLE. Neurological involvement in SLE patients is thought to be correlated with APL antibody [31, 32], but previous studies have not shown this association [28, 29], perhaps due to the low incidence of neuropsychological lupus in SLE patients. We demonstrated a tendency (*P* = 0.061) of association between APL antibody and neurological involvement in our study (Table 4). Further studies with more patients are needed.

Using cluster analysis, we identified five clusters of autoantibodies. Antibodies to Sm and RNP clustered together early. Cluster 2 consisted of antibodies to SSA and SSB. The other clusters consisted of individual antibodies to ribosomal P (rRNP), dsDNA, and APL, respectively (Figure 1). Our result is in accordance with previous studies [6, 33].

We summarize the associations with statistical significance between specific autoantibodies and clinical manifestations revealed by different study groups in Table 5. Most of our findings are consistent with studies in the literature, but the associations between anti-RNP antibody and PAH; between anti-SSA and PAH; and between APL antibody and ILD were first discovered by our study. We always repeated echocardiography if PAH was suspected in the first echocardiogram examination. Right heart catheter (RHC) is not feasible for general screening and repeated measurements due to its invasive characteristic but RHC is used for confirming the diagnosis of PAH according to the guideline. As far as we know, most of the 74 patients with a preliminary diagnosis of PAH identified in our study have been referred for confirmatory RHC, but these results were not recorded in the registry. This is a limitation of our study [34].

## 5. Conclusion 

As the largest registry cohort study in China, CSTAR has already disclosed some clinical profiles of Chinese SLE patient [8, 34]. Confirmation of the associations between clinical manifestations and specific autoantibodies found in our study can help physicians to understand the features of SLE patients better, especially in China. A thorough screening of ANA and anti-ENA antibodies when the diagnosis of SLE is established can help us to predict organ damage. We could focus on the specific autoantibody-related vital organ complications (e.g., PAH) in the follow-up of SLE patients. We suggest that repeating echocardiography annually may help to discover PAH at the early stage in SLE patients with anti-RNP and/or anti-SSA antibody. Early diagnosis of PAH in patients with SLE is important for initiating effective interventions to prevent malignant outcomes (e.g., heart failure).

## Figures and Tables

**Table 1:** The baseline characteristics of 2104 SLE patients from CSTAR cohort study.

		%
Female	1914	91.0
Male	190	9.0
Age at onset (years)	29.2 ± 12.1 (range 1.4~68.9)	
Age at diagnosis (years)	30.3 ± 12.3 (range 4~77)	
Age at entry (years)	32.7 ± 12.7 (range 5~78)	
Disease duration (months)	41.9 ± 58.8 (range 1~468)	
SLE disease activity index at entry		
0~4	532	25.3
5~9	587	27.9
10~14	591	28.1
>14	394	18.7

**Table 2:** The profile of autoantibodies in 2104 SLE patients from CSTAR cohort study.

	Patients number	Positivity (%)
Anti-nuclear antibody (ANA)	2063	98.1
Anti-double stranded DNA (anti-dsDNA) antibody	699	33.2
Anti-Sm antibody	350	16.6
Anti-SSA antibody	497	23.6
Anti-SSB antibody	224	10.7
Anti-u1 small-nuclear RNA-protein (anti-RNP) antibody	189	8.9
Anti-ribosomal RNA-protein (anti-rRNP) antibody	255	12.7
Anti-phospholipid (APL) antibody	414/937^$^	44.1
Anti-SSA and anti-SSB antibody positive simultaneously	199	9.5
Anti-Sm and anti-RNP antibody positive simultaneously	142	6.7

^$^Actually detected number of patients.

**Table 3:** The profile of clinical manifestations in 2104 SLE patients from CSTAR cohort study.

	Patients number	Positivity (%)
Malar rash	1009	47.9
Discoid lesions	118	5.6
Photosensitivity	526	25.0
Oral ulcerations	466	22.1
Arthritis	1147	54.5
Serositis	345	16.4
Nephropathy	998	47.4
Hematological involvement (hematocytopenia)	1181	56.1
Neurological involvement	101	4.8
Interstitial lung disease	86/2024^$^	4.2
Pulmonary arterial hypertension	74/1934^$^	3.8

^$^Actually detected number of patients.

**Table 4:** Associations between specific autoantibodies and clinical manifestations of SLE [patient number (%)].

	Anti-dsDNA		Anti-Sm		Anti-RNP		Anti-SSA		Anti-SSB		Anti-rRNP		APL	
	Positive	*P*	Positive	*P*	Positive	*P*	Positive	*P*	Positive	*P*	Positive	*P*	Positive	*P*
Patients number	699 (100)		350 (100)		189 (100)		497 (100)		224 (100)		255 (100)		414 (100)	
Malar rash	315 (45.06)	0.061	201 (57.43)	<0.001*	91 (48.15)	0.956	241 (48.49)	0.785	105 (46.88)	0.732	151 (59.22)	0.004*	187 (45.17)	0.275
Discoid lesions	31 (4.43)	0.099	22 (6.29)	0.546	13 (6.88)	0.426	25 (5.03)	0.522	11 (4.91)	0.631	13 (5.10)	0.855	20 (4.83)	0.540
Photosensitivity	150 (21.46)	0.008*	95 (27.14)	0.311	47 (24.87)	0.965	132 (26.56)	0.358	67 (29.91)	0.073	69 (27.06)	0.752	96 (23.19)	0.509
Oral ulcerations	151 (21.6)	0.671	87 (24.86)	0.181	42 (22.22)	0.980	108 (21.73)	0.797	48 (21.43)	0.784	48 (18.82)	0.395	74 (17.87)	0.035*
Arthritis	399 (57.08)	0.095	193 (55.14)	0.796	106 (56.08)	0.650	268 (53.92)	0.762	119 (53.13)	0.658	146 (57.25)	0.765	236 (57.00)	0.289
Serositis	119 (17.02)	0.584	55 (15.71)	0.705	31 (16.40)	0.999	89 (17.91)	0.298	46 (20.54)	0.077	35 (13.73)	0.428	61 (14.73)	0.996
Nephropathy	355 (50.79)	0.030*	155 (44.29)	0.196	91 (48.15)	0.837	216 (43.46)	0.042*	102 (45.54)	0.547	120 (47.06)	0.817	191 (46.14)	0.848
Hematological involvement	403 (57.65)	0.321	196 (56.00)	0.957	108 (57.14)	0.769	278 (55.94)	0.920	141 (62.95)	0.030*	137 (53.73)	0.542	273 (65.94)	<0.001*
Neurological involvement	26 (3.72)	0.102	18 (5.14)	0.743	12 (6.35)	0.296	21 (4.23)	0.493	13 (5.80)	0.457	16 (6.27)	0.335	32 (7.73)	0.061
ILD	29/675^$^ (4.30)	0.941	18/326^$^ (5.52)	0.214	11/181^$^ (6.08)	0.201	21/481^$^ (4.37)	0.884	12/209^$^ (5.74)	0.259	12/250^$^ (4.80)	0.940	29/398^$^ (7.29)	<0.001*
PAH	24/661^$^ (3.63)	0.747	16/322^$^ (4.97)	0.242	12/180^$^ (6.67)	0.037*	28/469^$^ (5.97)	0.005*	11/205^$^ (5.37)	0.224	5/237^$^ (2.11)	0.151	15/387^$^ (3.88)	0.714

*P*: *P* value; Anti-dsDNA: Anti-double stranded DNA antibody; Anti-Sm: Anti-Sm antibody; Anti-SSA: Anti-SSA antibody; Anti-SSB: Anti-SSB antibody; Anti-RNP: Anti-u1 small-nuclear RNA-protein antibody; Anti-rRNP: Anti-ribosomal RNA-protein antibody; APL: Anti-phospholipid antibody; PAH: pulmonary arterial hypertension; ILD: interstitial lung disease.

^$^Actually detected number of patients.

*P < 0.05.

**Table 5:** Associations with statistical significance between specific autoantibodies and clinical manifestations in SLE patients.

Author	Number of patients	Geographical area	Anti-dsDNA	Anti-Sm	Anti-RNP	Anti-SSA	Anti-SSB	Anti-rRNP	APL
Chien et al. [13]	80	Asia, China	Raynaud's phenomenon Photosensitivity Arthritis Thrombocytopenia Hypocomplementemia Proteinuria Serositis			Photosensitivity Anemia			

McClain et al. [28]	130	United states							Malar rash discoid lesions photosensitivity renal disorder neurological disorder hemolytic anemia thrombocytopenia

Vila et al. [24]	201	Puerto Ricans	Vasculitis Pericardial effusion Renal involvement Anaemia Leukocytopenia Lymphocytopenia Thrombocytopenia	Skin ulcerations Elevated liver enzymes Renal involvement Thrombocytopenia		Discoid rash Serositis Pneumonitis Elevated liver enzymes Hemolytic anaemia Leukocytopenia Lymphocytopenia			

Hoffman et al. [6]	289	Europe, Belgium	Urine cellular casts		Raynaud's phenomenon Leukocytopenia	Xerostomia	Xerostomia Pericarditis		

Tang et al. [7]	917	Asia, China	Renal disorder Leukocytopenia Anemia	Malar rash Discoid rash Pericarditis Leukocytopenia	Raynaud's phenomenon Photosensitivity				

Lu et al. [12]	1803	United states	Renal disease Urine cellular casts	Leukocytopenia		Hematological disorder Lymphocytopenia	Hematological disorder Proteinuria Malar rash		

CSTAR	2104	Asia, China	Photosensitivity Nephropathy	Malar rash	PAH	Nephropathy PAH	Hematological disorder	Malar rash	Oral ulceration hematological disorder ILD

ANA: Anti-nuclear antibody; Anti-dsDNA: Anti-double stranded DNA antibody; Anti-Sm: Anti-Sm antibody; Anti-SSA: Anti-SSA antibody; Anti-SSB: Anti-SSB antibody; Anti-RNP: Anti-u1 small-nuclear RNA-protein antibody; Anti-Rrnp: Anti-ribosomal RNA-protein antibody; APL: Anti-phospholipid antibody; PAH: pulmonary arterial hypertension; ILD: interstitial lung disease.

## Abbreviations

ANA: Anti-nuclear antibody
ENA: Extractable nuclear antigen
Anti-dsDNA: Anti-double stranded DNA antibody
Anti-Sm: Anti-Sm antibody
Anti-SSA: Anti-SSA antibody
Anti-SSB: Anti-SSB antibody
Anti-RNP: Anti-u1 small-nuclear RNA-protein antibody
Anti-rRNP: Anti-ribosomal RNA-protein antibody
APL: Anti-phospholipid antibody
PAH: Pulmonary arterial hypertension
ILD: Interstitial lung disease.
